# Protein arginine methyltransferase 3 inhibits renal tubulointerstitial fibrosis through asymmetric dimethylarginine

**DOI:** 10.3389/fmed.2022.995917

**Published:** 2022-09-13

**Authors:** Yanzhe Wang, Ming Wu, Feng Yang, Junyan Lin, Li Zhang, Meijie Yuan, Dongping Chen, Bo Tan, Di Huang, Chaoyang Ye

**Affiliations:** ^1^Department of Nephrology, Shuguang Hospital Affiliated to Shanghai University of Traditional Chinese Medicine, Shanghai, China; ^2^TCM Institute of Kidney Disease of Shanghai University of Traditional Chinese Medicine, Shanghai, China; ^3^Key Laboratory of Liver and Kidney Diseases, Ministry of Education, Shanghai Key Laboratory of Traditional Chinese Clinical Medicine, Shanghai, China; ^4^Department of Pediatrics, Shuguang Hospital Affiliated to Shanghai University of Traditional Chinese Medicine, Shanghai, China; ^5^Department of Nephrology, The First Hospital of Hebei Medical University, Shijiazhuang, China; ^6^Clinical Pharmacokinetic Laboratory, Shuguang Hospital Affiliated to Shanghai University of Traditional Chinese Medicine, Shanghai, China

**Keywords:** ADMA, fibrosis, arginine methyltransferase, chronic kidney disease, obstructive nephropathy

## Abstract

Mammalian protein arginine methyltransferase 3 (PRMT3) catalyzes the monomethylation and dimethylation of the arginine residues of proteins. The role of PRMT3 in renal fibrosis is currently unknown. We aimed to study the role of PRMT3 in renal fibrosis and explored its underlying mechanisms. Quantitative PCR analysis and Western blotting analysis showed that the expression of PRMT3 was up-regulated in unilateral ureteral obstruction (UUO) mouse kidneys. Knockout of *Prmt3* gene enhanced interstitial fibrosis in UUO kidneys as shown by Masson staining and Western blotting analysis the expression of pro-fibrotic markers. The production of asymmetric dimethylarginine (ADMA) was increased in wide type UUO kidneys but not further increased in *Prmt3* knockout UUO kidneys. Administration of exogeneous ADMA in UUO kidneys blocked the enhanced renal interstitial fibrosis in *Prmt3* mutant mice. Moreover, genetic deletion of *Prmt3* gene increased blood urea nitrogen levels and renal deposition of collagen in folic acid injected mice. We conclude that PRMT3 inhibits renal tubulointerstitial fibrosis through elevating renal ADMA levels.

## Introduction

Chronic kidney disease (CKD) is a leading public health problem caused by repeated injuries to glomerulus or renal tubules (1, 2). Renal tubulointerstitial fibrosis is the common pathway of all kinds of CKD progressing to the end-stage renal disease regardless its causes (3, 4). Histologically, it is characterized by excessive deposition of extracellular matrix proteins such as collagen-I and overexpression of epithelial mesenchymal transition markers (e.g., α-SMA) in renal interstitial areas leading to completely loss of renal function (5, 6). Several animal models are used to study renal interstitial fibrosis which mimic obstructive or toxic injuries such as unilateral ureteral obstruction (UUO) or folic acid (FA) induced injuries in kidneys (7, 8).

Arginine methylation is a research focus in the field of post-translational modification which is involved in various biological processes (9). Nine protein arginine methyltransferases (PRMTs) have been identified in mammalian cells and are divided into three categories (9). PRMT1, 2, 3, 4, 6, and 8 belong to type I PRMTs, which can catalyze the methylation at arginine residues to form monomethylation (MMA) and asymmetric dimethylarginine (ADMA) (9, 10). We previously showed that inhibition of type I PRMTs promoted renal fibrosis in the UUO mouse model suggesting that type I PRMTs are renal protective (11). However, a recent study indicated that PRMT1 is pro-fibrotic in the UUO model by using a selective inhibitor implying that other member of type I PRMT family is anti-fibrotic in kidney diseases (12). PRMT3 is a member of type I methyltransferase, which is mainly located in the cytoplasm and modifies ribosomal proteins (9). Recently it is found that PRMT3 interacts and modifies the activity of many enzymes involving different biological processes (13–15). However, the role of PRMT3 in renal fibrosis is currently unknown.

In the present study, we aimed to determine the role of PRMT3 in renal tubulointerstitial fibrosis and explore its underlying mechanism.

## Results

### Protein arginine methyltransferase 3 inhibits renal tubulointerstitial fibrosis in obstructed mouse kidneys

**FIGURE 1:**
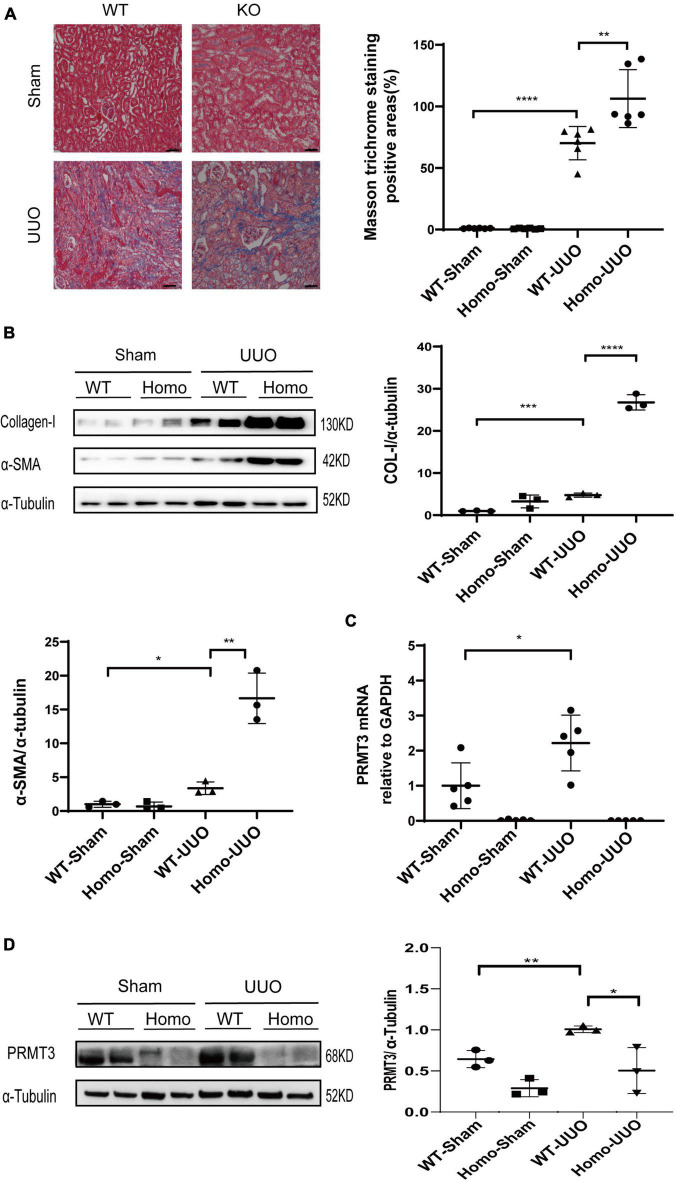
Knockout of *Prmt3* promotes renal fibrosis in obstructed mouse kidneys. Wide type (WT) or homozygous (Homo) *Prmt3* mutant c57 mice received sham or UUO operation and were sacrificed at day 14 (*n* = 9–10 mice per group). **(A)** Renal fibrosis was assessed by Masson’s trichrome staining (*n* = 6–7 mice per group). Bars, 100 μm. **(B)** The expression of α-SMA and collagen-I were analyzed by Western blotting. **(C,D)** The expression of PRMT3 were analyzed by qPCR or Western blotting. One representative of at least three independent experiments is shown. Data represent mean ± SD. One-way ANOVA was used and comparison between two groups was performed by unpaired student *t*-test. *P < 0.05; **P < 0.01; ***P < 0.001; ****P < 0.0001.

The role of PRMT3 in renal fibrosis was studied *in vivo* in the UUO mouse model using *Prmt3* gene knockout mice. The birth rate of homozygous (Homo) *Prmt3* knockout mice is low (less than 10% of pups). *Prmt3* Homo knockout mice have low body weight after weaning but can catch up normal body weight in adulthood. Moreover, no interstitial collagen deposition was observed in Homo *Prmt3* knockout sham mice as shown by Masson staining, suggesting a normal development of kidney after *Prmt3* gene deletion (Figure 1A).

A significant increase in Masson positive staining areas was observed in WT UUO kidneys, which was further increased in Homo *Prmt3* UUO kidneys (shown in Figure 1A). The protein expression of α-SMA and collagen-I were up-regulated in WT UUO mouse kidneys as compared with that in WT sham operated mouse kidneys, and homozygous deletion of *Prmt3* significantly increased the expression of α-SMA and collagen-I in UUO mouse kidneys (shown in Figure 1B). The expression of renal PRMT3 was measured by qPCR and Western blotting. As shown in Figure 1C, mRNA levels of PRMT3 were significantly up-regulated in WT UUO kidneys as compared with that in WT sham kidneys, which was reduced in Homo *Prmt3* UUO kidneys. Similarly, the expression of PRMT3 protein was increased after UUO operation in WT kidneys (Figure 1D). The expression of PRMT3 protein was abolished in *Prmt3* genetic knockout sham or UUO kidneys (Figure 1D).

### Histone arginine methylation but not asymmetric dimethylarginine production is increased in fibrotic kidneys after *Prmt3* deletion

**FIGURE 2:**
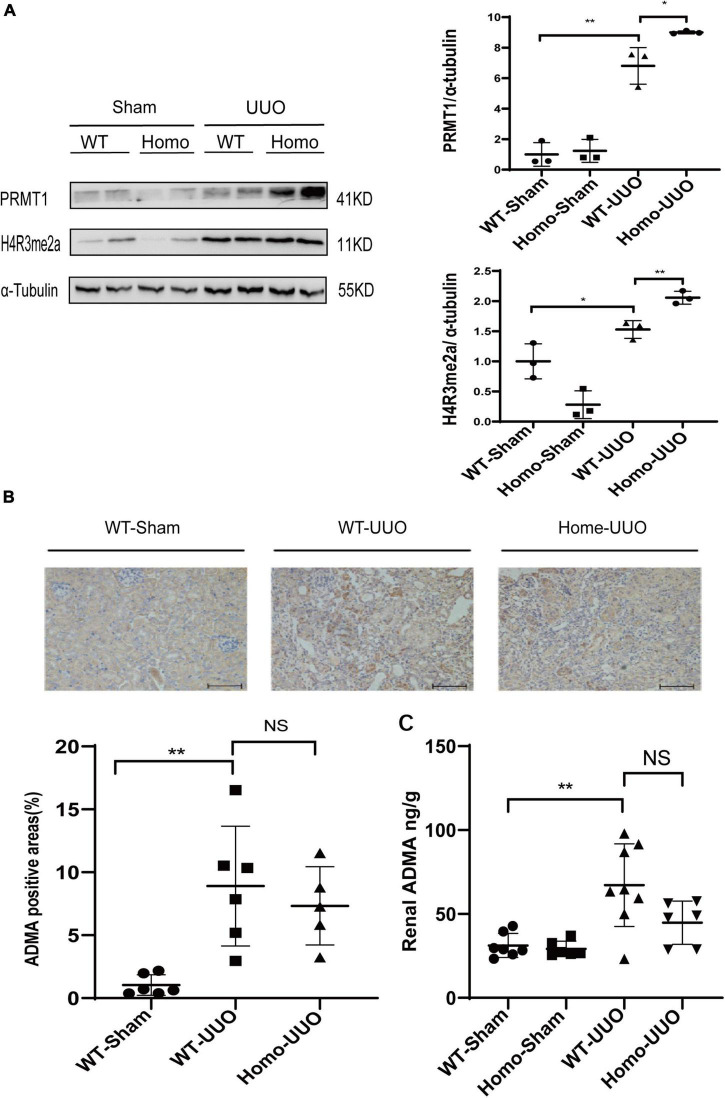
Changes of histone methylation and ADMA production in fibrotic kidneys after *Prmt3* deletion. **(A)** The expression of PRMT1 and H4R3me2a in WT or *Prmt3* Homo mutant c57 mice were analyzed by Western blotting and quantified; **(B,C)** renal ADMA accumulation in WT or *Prmt3* Homo mutant c57 mice were analyzed by immunohistochemistry (*n* = 5–6 mice per group) and ELISA (*n* = 5–8 mice per group). Bars, 100 μm. Data represent mean ± SD. One-way ANOVA was used and comparison between two groups was performed by unpaired student *t*-test. NS represent not significant; *P < 0.05; **P < 0.01. One representative result of at least three independent experiments is shown.

The enzymatic activity of PRMT3 was assessed by measuring asymmetric dimethylation of histone H4 arginine 3 (H4R3me2a) or production of (ADMA in kidneys. The expression of H4R3me2a was increased in WT UUO kidneys as compared to that in WT Sham kidneys as shown by Western blotting, and it was further increased in Homo *Prmt3* UUO kidneys (Figure 2A). The expression of PRMT1, an upstream enzyme of H4R3me2a, was also increased in WT UUO kidneys as compared with that in WT Sham kidneys (Figure 2A). There was a further increase in PRMT1 expression in UUO kidneys after *Prmt3* homozygous deletion (Figure 2A).

Increased cytoplasmic accumulation of ADMA was observed in tubules of WT UUO kidneys as compared with that in WT Sham kidneys as shown by immunohistochemistry staining, and there was no further increase in ADMA staining in tubules of Homo *Prmt3* UUO kidneys (Figure 2B). Renal ADMA levels were further determined by the ELISA assay, which showed an increased renal ADMA content after UUO operation in WT kidneys and no further changes of ADMA content after *Prmt3* homozygous deletion in UUO kidneys (Figure 2C).

### Exogenous asymmetric dimethylarginine mitigates fibrosis in unilateral ureteral obstruction kidneys after *Prmt3* heterozygous deletion

**FIGURE 3:**
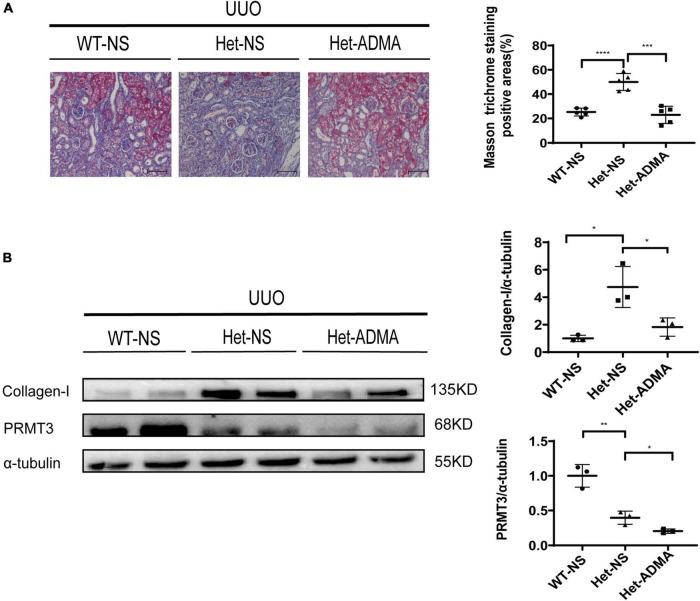
Asymmetric dimethylarginine mitigates fibrosis in *Prmt3* deleted UUO kidneys. WT or heterozygous (Het) *Prmt3* mutant c57 mice received sham or UUO operation and were sacrificed at day 7 (*n* = 7, WT-NS; *n* = 9, Het-NS; *n* = 8, Het-ADMA). Renal injection of normal saline (NS) or ADMA was performed during UUO operation. **(A)** Renal fibrosis was assessed by Masson’s trichrome staining (*n* = 5 mice per group). Bars, 100 μm. **(B)** The expression of collagen-I and PRMT3 were analyzed by Western blotting. Data represent mean ± SD. One-way ANOVA was used and comparison between two groups was performed by unpaired student *t*-test. *P < 0.05; **P < 0.01; ***P < 0.001; ****P < 0.0001. One representative result of at least three independent experiments is shown.

**FIGURE 4:**
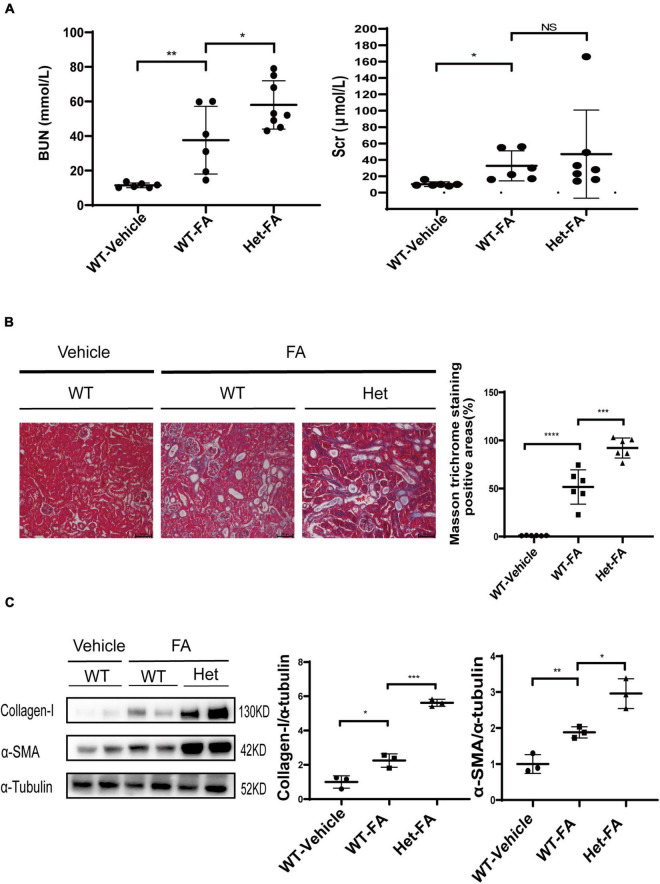
Heterozygous deletion of *Prmt3* exacerbates renal injuries in FA nephropathy. Mouse FA nephropathy was induced by intraperitoneal injection of FA (250 mg/kg in 150 mmol/L sodium carbonate) in WT or Het *Prmt3* mutant c57 mice, and mice were sacrificed at 48 h (*n* = 6, WT-vehicle; *n* = 6, WT-FA; *n* = 8, Het-FA). **(A)** Renal function (blood urea nitrogen, BUN; serum creatinine, Scr) was assessed. Bars, 100 μm. **(B)** Renal fibrosis was assessed by Masson’s trichrome staining (*n* = 6 mice per group). **(C)** The expression of α-SMA and collagen-I were analyzed by Western blotting. One representative of at least three independent experiments is shown. Data represent mean ± SD. One-way ANOVA was used and comparison between two groups was performed by unpaired student *t*-test. NS represent not significant; *P < 0.05; **P < 0.01; ***P < 0.001; ****P < 0.0001. One representative result of at least three independent experiments is shown.

Normal saline or exogenous ADMA was delivered to UUO kidneys through retrograde ureteral injection. Renal fibrosis was assessed 1 week after UUO operation by Masson staining or Western blotting. *Prmt3* heterozygous (Het) deletion increased Masson positive staining areas and the expression of collagen-I at 1 week after UUO operation, and addition of exogenous ADMA reduced positive areas of Masson staining and expression of collagen-I in Het *Prmt3* knockout kidneys (Figures 3A, 4B). The expression of PRMT3 was reduced in Het Prmt3 knockout kidneys as compared with that in WT kidneys (Figure 3B).

### Heterozygous deletion of *Prmt3* exacerbates renal injuries in folic acid induced nephropathy

The role of PRMT3 was further studied in a mouse model of FA nephropathy. Mouse FA nephropathy was induced by intraperitoneal injection of FA (250 mg/kg in 150 mmol/L sodium carbonate), and mice were sacrificed at 48 h. As shown in Figure 4A, blood urea nitrogen (BUN) and serum creatinine (Scr) levels were elevated in WT FA mice as compared with that in WT vehicle mice, and heterozygous deletion of *Prmt3* significantly increased BUN but not Scr levels in FA mice. Masson staining showed a mild tubulointerstitial fibrosis in WT FA kidneys and heterozygous deletion of *Prmt3* gene significantly increased Masson positive staining areas in FA kidneys (Figure 4B). Western blotting analysis showed that collagen-I and α-SMA were slightly increased in WT FA kidneys as compared with that in WT vehicle kidneys, and heterozygous *Prmt3* gene deletion enhanced the expression of collagen-I and α-SMA (Figure 4C).

## Discussion

We previously showed that inhibition of type I PRMTs promotes renal fibrosis (11). To find out which family member of type I PRMTs is renal protective, we initiated the current study to investigate the role of PRMT3 in renal fibrosis. In the UUO mouse model, we found that homozygous deletion of *Prmt3* gene promoted renal fibrosis as shown by Masson staining and Western blotting analysis. The renal protective effect of PRMT3 was further confirmed in FA induced nephropathy by demonstrating that heterozygous deletion of Prmt3 increased BUN levels, collagen deposition and fibrotic marker expression. Thus, we conclude that PRMT3 is protective against renal fibrosis.

Protein arginine methyltransferase 3-mediated epigenetic regulation depends on arginine methylation on histone (9). Type I PRMTs catalyze H4R3 methylation, which is enhanced in fibrotic kidneys (11, 12, 16). In the current study, we first measured the expression of H4R3me2a in UUO kidneys, which is positively correlated with the expression of PRMT3 in WT UUO kidneys. However, genetic deletion of *Prmt3* in UUO kidneys did not reduce the expression of H4R3me2a instead of increased its expression. PRMT3 is mainly located in the cytoplasm and thus the downstream effect of PRMT3 in UUO kidneys is not through H4R3me2a-mediated epigenetic gene regulation (9, 17). PRMT1 is the main type I PRMTs responsible for histone methylation and gene transcriptional activation, which is upregulated in fibrotic kidneys (9, 12). Thus, the increased expression of H4R3me2a in *Prmt3* knockout UUO kidneys is probably the consequence of PRMT1 upregulation. Indeed, we observed the positive correlation of H4R3me2a expression with PRMT1 in WT and *Prmt3* knockout UUO kidneys.

Asymmetric dimethylarginine is a well-known risk factor of cardiovascular disease in patients with ESRD (16). However, recent study indicates that local production of ADMA in the kidney is beneficial to renal disease by using two renal fibrosis mouse models with kidney specific deletion of methylarginine-metabolizing enzyme dimethylaminohydrolase-1 (16). We further proved the protective effect of renal ADMA by using a type I PRMT inhibitor in UUO mouse model or direct injection of ADMA to UUO kidneys, which was also confirmed *in vitro* (11, 18). Therefore, we hypothesized that the enhanced fibrosis in *Prmt3* deleted UUO kidneys was due to an insufficiency of ADMA production. To prove this, we first measured the production of ADMA in kidneys. By immunohistochemistry staining and ELISA, we found that renal ADMA levels were increased after UUO operation, which were not further increased in *Prmt3* knockout mice and even slightly reduced but without statistical significance. Furthermore, delivery of exogeneous ADMA into UUO kidneys with *Prmt3* deletion attenuated renal fibrosis in *Prmt3* knockout UUO kidneys.

Recent studies show that PRMT3 exerts biological functions through interacting and regulating the activity of downstream transcriptional factors or enzymes (9, 13–15). Besides the general product of ADMA, PRMT3 may inhibit renal fibrosis through modification of certain transcriptional factors or enzymes. Further study with LC–MS/MS analysis should be performed to search PRMT3 associated proteins in renal fibrotic cells.

In conclusion, PRMT3 inhibits renal tubulointerstitial fibrosis through production of renal ADMA.

## Materials and methods

### Animal models

Wide type (WT) male C57 mice (SPF grade, 20–25 g) were purchased from Shanghai Model Organisms Center Inc. (SMOC), and *Prmt3* knockout mice with C57 BL/6 background were bought from Cyagen Biosciences (Suzhou, China). Animals were housed in the animal facility of Shanghai University of Traditional Chinese Medicine according to local regulations and guidelines. Animal experiments were approved by the ethic committee of Shanghai University of Traditional Chinese Medicine (PZSHUTCM18111601).

Unilateral ureteral obstruction model was established with 6–10-week-old mice as described before (11). In brief, UUO operation was performed through twice ligations of the left ureter with 4-0 nylon sutures. Animals were sacrificed at day 7 or day 14 according to the experimental design. Kidney samples were obtained for Western blotting analysis or histological examinations.

Since homozygous *Prmt3* knockout mice have a low birth rate (less than 10% of pups), only heterozygous *Prmt3* knockout mice were used in the following animal experiments. To establish FA-induced nephropathy, 6–8-week-old mice were injected intraperitoneally with FA (F7876, Sigma-Aldrich, dissolved in 0.3 M sodium bicarbonate at a dose of 250 mg/kg). Sodium bicarbonate alone was used as controls. At 48 h after FA administration, animals were sacrificed. Serum and kidney samples were collected for renal function or protein analysis.

For intra-renal injection, (ADMA (C5216) was bought from APExBIO (Houston, TX, United States) and dissolved in normal saline, and 0.04% trypan blue dye (A601140, Sangon, Shanghai, China) was added into ADMA solution for monitoring the injection process. A total of 50 μl of normal saline or ADMA (48 mg/ml) was injected retrogradely once into the left kidney *via* the ureter, which was followed by UUO surgery. Mice were sacrificed at day 7 for kidney sample collection.

### Enzyme-linked immunosorbent assay

Snap frozen kidney tissues were weighed, resuspended in 1:50 (w/v) to lysis buffer (IS007, Cloud-Clone Corp., Wuhan, China) and disrupted with a tissue homogenizer. ADMA levels in kidney homogenates were determined using an ELISA kit (Cloud-Clone Corp., Cat. No. ceB301Ge, Wuhan, China). All procedures of enzyme-linked immunosorbent assay (ELISA) were performed by Cloud-Clone Corp., Wuhan, China. For normalization, total protein in samples was estimated by the bicinchoninic acid (BCA) assay (Beyotime, P0011, Shanghai, China).

### Masson’s trichrome and immunohistochemistry staining

Mouse kidneys were fixed in 4% paraformaldehyde and embedded in paraffin. For masson’s trichrome staining, 3-μm-thick renal sections were stained with hematoxylin, and then with ponceau red liquid dye acid complex, which was followed by incubation with phosphomolybdic acid solution. Finally, sections were stained with aniline blue liquid and acetic acid. Images were captured using a microscope (Nikon Eclipse 80i, Tokyo, Japan). For immunohistochemistry (IHC) staining of ADMA (1:200, LSBio, LS-C295822/143216, Seattle, WA, United States), sections were routinely dewaxed and rehydrated. Antigen retrieval was performed in an autoclave oven with EDTA buffer at pH 8.0. Hydrogen peroxide was utilized to block endogenous peroxidase activity, and normal sheep serum was used to seal up non-specific binding sites. The primary antibody was incubated overnight at 4°C, followed by incubation with a horseradish peroxidase (HRP) labeled secondary antibody for 50 min at room temperature. Renal sections were stained with DAB, counterstained with hematoxylin, and mounted for further assessment. All the procedures were performed by Shanghai Ruchuang Biotechnology Company.

### Quantitative real-time PCR

Total RNA was extracted using TRUzol (R401-01, Vazyme, Nanjing, China) from kidney samples according to the manufacture’s instruction, which was reverse transcribed to cDNA by Takara PrimeScript RT reagent kit (RR0036A, Kyoto, Japan). Quantitative PCR was performed on an ABI StepOnePlus Real-Time PCR System (Applied Biosystems, Foster City, CA, United States) with SYBR Green Master Mix (Yeasen, Shanghai, China) according to the protocol from the manufacturer. Relative fold changes of gene expression were determined by the 2^–ΔΔCT^ method. Gapdh was used as an internal control. The PCR reaction conditions were as follows: 95°C for 5 min, followed by 40 cycles for 10 s at 95°C and 30 s at 60°C. The primer sequences for qPCR were listed as follows: mouse *Prmt3* forward, 5′-TGTGACAGGTTGTTCGCCT-3′; mouse *Prmt3* reverse, 5′-TTTCCCAAGGCACAGGGTTAT-3′; mouse *Gapdh* forward, 5′-AGGTCGGTGTGAACGGATTTG-3′; mouse *Gapdh* reverse, 5′-TGTAGACCATGTAGTTGAGGTCA-3′.

### Western blotting analysis

Protein was extracted from the medulla and cortex of mouse kidneys. The protein concentration was measured by the BCA method, and the supernatant was resuspended in 5× SDS-PAGE loading buffer (P0015L, Beyotime Biotech, Nantong, China). Samples were subjected to SDS-PAGE gels. After electrophoresis, proteins were electro-transferred to a polyvinylidene difluoride membrane (Merck Millipore, Darmstadt, Germany), which was incubated in the blocking buffer (5% non-fat milk, 20 mM Tris–HCl, 150 mM NaCl, PH = 8.0, 0.01% Tween-20) for 1 h at room temperature and was followed by incubation with anti-Collagen-I (1:500, sc-293182, Santa Cruz), anti-PRMT3 (1:1,000, A13068, Abclonal), anti-PRMT1 (1:1,000, 2449S, Cell Signaling Technology), anti- H4R3me2a (1:1,000, SAB4300868, Sigma), or anti-α-tubulin (1:1,000, AF0001, Byotime) antibodies overnight at 4°C. Binding of the primary antibody was detected by an enhanced chemiluminescence method (34094, SuperSignal*™* West Femto, Thermo Fisher Scientific) using HRP-conjugated secondary antibodies (goat anti-rabbit IgG, 1:1,000, A0208, Beyotime or goat anti-mouse IgG, 1:1,000, A0216, Beyotime). The quantification of protein expression was performed using ImageJ (National Institute of Health, Bethesda, MD, United States).

### Statistical analysis

Statistical analysis was performed by GraphPad Prism version 8.0.0 for Windows (GraphPad Software, San Diego, CA, United States). Results were showed as mean ± SD. Differences among multiple groups were analyzed by one-way analysis of variance (ANOVA) and comparison between two groups was performed by unpaired student *t*-test. A *P*-value of lower than 0.05 was considered statistically significant.

## Data availability statement

The original contributions presented in this study are included in the article/supplementary material, further inquiries can be directed to the corresponding authors.

## Ethics statement

The animal study was reviewed and approved by the Shanghai University of Traditional Chinese Medicine.

## Author contributions

MW conceived and coordinated the study and wrote the manuscript. MW, YW, FY, JL, DC, BT, and DH performed the animal experiments. YW and LZ performed and analyzed the Western blotting. All authors reviewed the results and approved the final version of the manuscript.

## References

[1] Irazabal MV Torres VE. Reactive oxygen species and redox signaling in chronic kidney disease. *Cells.* (2020) 9:1342. 10.3390/cells9061342 PMC7349188 32481548

[2] Shabaka A Cases-Corona C Fernandez-Juarez G. Therapeutic insights in chronic kidney disease progression. *Front Med (Lausanne).* (2021) 8:645187. 10.3389/fmed.2021.645187 33708784 PMC7940523

[3] Humphreys BD. Mechanisms of Renal Fibrosis. *Annu Rev Physiol.* (2018) 80:309–26. 29068765 10.1146/annurev-physiol-022516-034227

[4] Tang PM Zhang YY Mak TS Tang PC Huang XR Lan HY. Transforming growth factor-beta signalling in renal fibrosis: from Smads to non-coding RNAs. *J Physiol.* (2018) 596:3493–503. 29781524 10.1113/JP274492 PMC6092283

[5] Gewin L Zent R Pozzi A. Progression of chronic kidney disease: too much cellular talk causes damage. *Kidney Int.* (2017) 91:552–60. 10.1016/j.kint.2016.08.025 27773427 PMC5313325

[6] Hewitson TD Holt SG Smith ER. Progression of tubulointerstitial fibrosis and the chronic kidney disease phenotype - role of risk factors and epigenetics. *Front Pharmacol.* (2017) 8:520. 10.3389/fphar.2017.00520 28848437 PMC5550676

[7] Chevalier RL Forbes MS Thornhill BA. Ureteral obstruction as a model of renal interstitial fibrosis and obstructive nephropathy. *Kidney Int.* (2009) 75:1145–52. 10.1038/ki.2009.86 19340094

[8] Fu Y Tang C Cai J Chen G Zhang D Dong Z. Rodent models of AKI-CKD transition. *Am J Physiol Renal Physiol.* (2018) 315:F1098–106. 29949392 10.1152/ajprenal.00199.2018 PMC6230729

[9] Blanc RS Richard S. Arginine methylation: the coming of age. *Mol Cell.* (2017) 65:8–24. 28061334 10.1016/j.molcel.2016.11.003

[10] Wang C Jiang H Jin J Xie Y Chen Z Zhang H Development of potent type i protein arginine methyltransferase (PRMT) inhibitors of leukemia cell proliferation. *J Med Chem.* (2017) 60:8888–905. 29019697 10.1021/acs.jmedchem.7b01134

[11] Wu M Lin P Li L Chen D Yang X Xu L Reduced asymmetric dimethylarginine accumulation through inhibition of the type I protein arginine methyltransferases promotes renal fibrosis in obstructed kidneys. *FASEB J.* (2019) 33:6948–56. 10.1096/fj.201802585RR 30840839

[12] Zhu Y Yu C Zhuang S. Protein arginine methyltransferase 1 mediates renal fibroblast activation and fibrogenesis through activation of Smad3 signaling. *Am J Physiol Renal Physiol.* (2020) 318:F375–87. 10.1152/ajprenal.00487.2019 31813251 PMC7052653

[13] Huang L Wang Z Narayanan N Yang Y. Arginine methylation of the C-terminus RGG motif promotes TOP3B topoisomerase activity and stress granule localization. *Nucleic Acids Res.* (2018) 46:3061–74. 10.1093/nar/gky103 29471495 PMC5888246

[14] Hsu MC Pan MR Chu PY Tsai YL Tsai CH Shan YS Protein arginine methyltransferase 3 enhances chemoresistance in pancreatic cancer by methylating hnRNPA1 to Increase ABCG2 Expression. *Cancers (Basel).* (2018) 11:8. 10.3390/cancers11010008 30577570 PMC6356582

[15] Verma M Khan MIK Kadumuri RV Chakrapani B Awasthi S Mahesh A PRMT3 interacts with ALDH1A1 and regulates gene-expression by inhibiting retinoic acid signaling. *Commun Biol.* (2021) 4:109. 10.1038/s42003-020-01644-3 33495566 PMC7835222

[16] Tomlinson JA Caplin B Boruc O Bruce-Cobbold C Cutillas P Dormann D Reduced renal methylarginine metabolism protects against progressive kidney damage. *J Am Soc Nephrol.* (2015) 26:3045–59. 10.1681/ASN.2014030280 25855779 PMC4657823

[17] Bachand F Silver PA. PRMT3 is a ribosomal protein methyltransferase that affects the cellular levels of ribosomal subunits. *EMBO J.* (2004) 23:2641–50. 10.1038/sj.emboj.7600265 15175657 PMC449775

[18] Wu M Yuan M Wang Y Tan B Huang D Wang C Renal asymmetric dimethylarginine inhibits fibrosis. *FEBS Open Bio.* (2020) 10:2003–9. 10.1002/2211-5463.12949 PMC7530377 32794631

